# Mixed-valence molybdenum oxide as a recyclable sorbent for silver removal and recovery from wastewater

**DOI:** 10.1038/s41467-023-37143-2

**Published:** 2023-03-13

**Authors:** Penghui Shao, Ziwen Chang, Min Li, Xiang Lu, Wenli Jiang, Kai Zhang, Xubiao Luo, Liming Yang

**Affiliations:** 1 National-Local Joint Engineering Research Center of Heavy Metals Pollutants Control and Resource Utilization, Nanchang Hangkong University, 330063 Nanchang, P. R. China; 2 Department of Chemical Engineering, Chongqing University of Science and Technology, 401331 Chongqing, P. R. China; 3 Key Laboratory of Environmental Biotechnology, Research Center for Eco-Environmental Sciences, Chinese Academy of Sciences, 100085 Beijing, P. R. China

**Keywords:** Pollution remediation, Sustainability, Materials chemistry

## Abstract

Silver ions in wastewater streams are a major pollutant and a threat to human health. Given the increasing demand and relative scarcity of silver, these streams could be a lucrative source to extract metallic silver. Wastewater is a complex mixture of many different metal salts, and developing recyclable sorbents with high specificity towards silver ions remains a major challenge. Here we report that molybdenum oxide (MoO_x_) adsorbent with mixed-valence (Mo(V) and Mo(VI)) demonstrates high selectivity (distribution coefficient of 6437.40 mL g^−1^) for Ag^+^ and an uptake capacity of 2605.91 mg g^−1^. Our experimental results and density functional theory calculations illustrate the mechanism behind Ag^+^ adsorption and reduction. Our results show that Mo(V) species reduce Ag^+^ to metallic Ag, which decreases the energy barrier for subsequent Ag^+^ reductions, accounting for the high uptake of Ag^+^ from wastewater. Due to its high selectivity, MoO_x_ favorably adsorbs Ag^+^ even in the presence of interfering ions. High selective recovery of Ag^+^ from wastewater (recovery efficiency = 97.9%) further supports the practical applications of the sorbent. Finally, MoO_x_ can be recycled following silver recovery while maintaining a recovery efficiency of 97.1% after five cycles. The method is expected to provide a viable strategy to recover silver from wastewater.

## Abstract (web summary)

Silver ions in wastewater are a major health hazard, and there is an urgent need to develop methods to reliably extract them. Here, the authors show that mixed-valence molybdenum oxide can selectively adsorb and reduce Ag^+^ from wastewater and be easily recycled while maintaining high selectivity towards Ag^+^.

## Introduction

Silver (Ag) is among the most important precious metals and has been widely used in various industrial fields, especially the electroplating industry^1^. Massive amounts of Ag^+^-containing electroplating wastewater are discharged; thus, toxic Ag species are inevitably released into the aquatic environment, causing a large potential risk to the ecological environment and human health^2,3^. In addition, increasing demand for Ag in various fields has caused a crisis, as Ag resources are being depleted^4,5^. Thus, the ability to extract precious Ag from wastewater is of great significance. Numerous technologies have been devoted to recovering Ag^+^, such as electrochemical deposition^6^, membrane separation^7^, biological treatment^8^, and adsorption^9^. As an economically feasible method, adsorption has attracted significant attention in the remediation of Ag-polluted wastewater^10,11^. Much effort has been dedicated to developing adsorbents with high capacity and excellent selectivity^12–15^. However, due to the interference of coexisting metal ions, the strong acidity of actual Ag^+^-polluted water, and the widespread dissolved humic acid in the water environment, recovering Ag^+^ is extremely difficult^16–20^. Furthermore, the complexity of actual Ag^+^-polluted water has barely been considered in the design and development of Ag^+^-adsorbents. As a result, the high-performance adsorbents developed in the laboratory are less effective in practical remediation processes.

To selectively recover Ag^+^, considerable effort has been made to establish the specific interaction between Ag^+^ and adsorbents, such as ion-imprinting adsorbents and sulfur-rich adsorbents^21,22^. The former method involves selectively adsorbing Ag^+^ by constructing specific cavities that match Ag^+^, while the latter method utilizes the very strong ability of sulfur to bind Ag^+^, which can be attributed to the Lewis soft-soft interactions. Although these materials exhibit excellent selectivity for Ag^+^ adsorption, the following shortcomings remain: (1) To achieve the recovery of Ag^+^, these adsorbents need to elute Ag^+^ through desorbents (such as acid, alkali, or organic solution) or incinerate the adsorbents after adsorption. However, the eluents used cause complicated post-processing procedures and may lead to secondary pollution. Incineration is problematic as the process consumes much energy and generates waste gas. (2) The service life of the adsorbent is also a major obstacle that restricts the application of these materials in actual Ag^+^-containing wastewater. These adsorbents turn into waste products after use, which not only increases the costs of Ag^+^ recovery but also contradicts the concept of sustainable development. Therefore, designing a recyclable waste-free adsorbent with the ability to selectively recover Ag^+^ is a great challenge.

Compared to most metal ions, Ag^+^ possesses a relatively high redox potential (0.80 V vs. SHE, Supplementary Fig. 1)^23,24^ which endows Ag^+^ with deposition ability on a special reductive adsorbent. Amorphous molybdenum oxide (MoO_x_), as an excellent photo/electro-catalyst, possesses great electron mass transfer capability^25–28^. The amorphous structure of MoO_x_ allows it to expose more active sites, which is beneficial to the adsorption of heavy metal ions. Moreover, due to the presence of low-valent Mo in the material, MoO_x_ exhibits a mild reduction performance. Therefore, it is feasible to recover Ag^+^ through a spontaneous redox reaction using MoO_x_ with a mixed valence. In addition, it was found that molybdenum oxide in an ammonia solution could be transformed into ammonium molybdate, which is the raw material for the synthesis of MoO_x_. This inspired us to design a subversive adsorbent recycling strategy.

Herein, we successfully designed and synthesized an amorphous MoO_x_ with reductive Mo(V) based on a redox precipitation mechanism using an electrochemical technique. Then, batch Ag^+^-recovery experiments were performed to evaluate the performance of amorphous MoO_x_. Through experimental analysis and density functional theory (DFT) calculations, we also fundamentally elucidated the mechanisms of MoO_x_ capture Ag^+^. Moreover, a flow-through reactor was designed to evaluate the Ag recovery and demonstrate the superior application potential of MoO_x_ to recover metallic Ag from actual Ag^+^-containing wastewater. In addition, a closed-loop recycling method to recover Ag^+^ and regenerate MoO_x_ was tested. Finally, we evaluated the regeneration performance of MoO_x_ and considered the economic benefits of MoO_x_ recovery Ag to further demonstrate the potential for practical application.

## Results

### Characterization of amorphous MoO_x_

Amorphous MoO_x_ was synthesized and loaded onto the F-doped tin oxide (FTO) surface by a simple one-step cyclic voltammetry (CV) electrodeposition method. In contrast to pale green bare FTO (Supplementary Fig. 4a), electrodeposited FTO is covered with a brown MoO_x_ film. Sheet-like MoO_x_ with a smooth surface grows uniformly on the FTO surface (Supplementary Fig. 4b). Energy dispersive spectroscopy (EDS) mapping analysis shows that O and Mo are evenly distributed on the surface (Supplementary Fig. 5b, c). The specific surface area of amorphous MoO_x_ is as low as 17.6 m^2^ g^−1^ (Supplementary Fig. 6a), and the pore size is mainly less than 5 nm, showing a low porosity structure. In the XRD pattern (Supplementary Fig. 6b), all peaks appeared with small peak intensities and wide peak widths, exhibiting an amorphous structure^29^, which was also confirmed by the selected area electron diffraction (SAED) pattern (inset of Supplementary Fig. 6b). Compared with regularly arranged crystalline structures, amorphous MoO_x_ possesses more unsaturated or defective atoms^30^, which are more conducive to capturing heavy metal ions. Furthermore, to ascertain the chemical state of Mo, the corresponding high-resolution Mo 3*d* XPS spectra were analyzed (Supplementary Fig. 6c). Both deconvoluted peaks centered at 231.70 eV and 234.82 eV are assigned to Mo(V), and the other two peaks at 232.70 eV and 235.88 eV correspond to Mo(VI)^31,32^. Impressively, the relative content of Mo(V) is up to 71.9%, while relatively stable Mo(VI) occupies 28.1%. A large amount of Mo(V) atoms is considered to enable the amorphous MoO_x_ equipped with an excellent redox ability^33,34^.

### Ag^+^ uptake capacity and selectivity

**Fig. 1:**
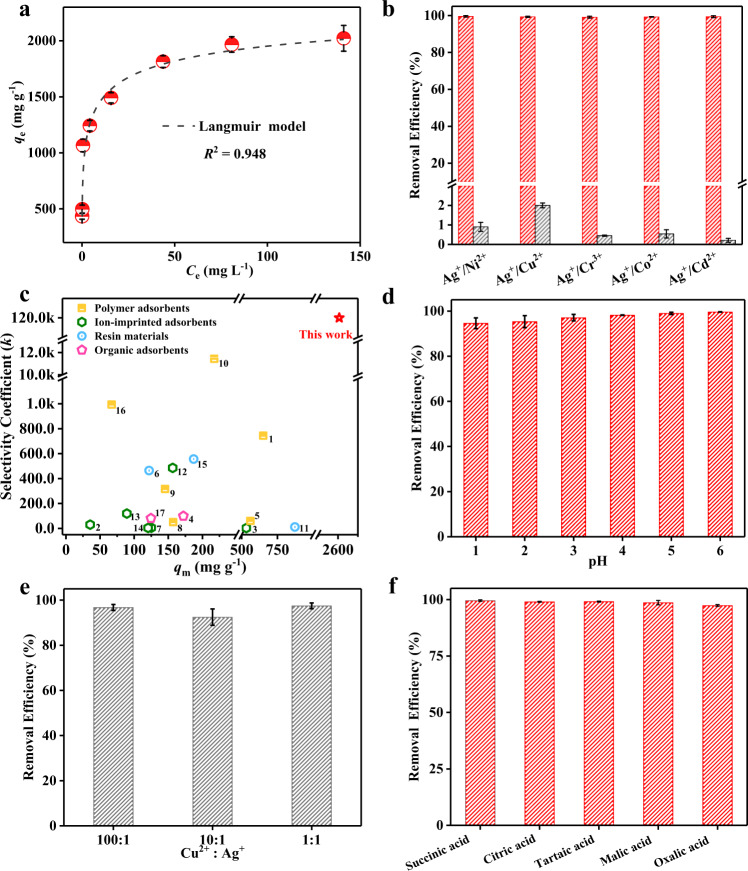
Ag^+^ adsorption and anti-interference performance of MoO_x_. **a** Adsorption isotherm of amorphous MoO_x_ towards Ag^+^ (initial Ag^+^ concentration was in the range of 10–250 mg L^−1^, solution volume was 100 mL, and pH was 5.0). The dashed line shows the fitting data of Langmiur model. The functional form and fitting parameters refer to Supplementary Table 2. **b** Removal efficiency for Ag^+^, Ni^2+^, Cu^2+^, Cr^3+^, Co^2+^, and Cd^2+^ in multi-metal and binary-metal (inset) mixed solutions (initial concentration of all metal ions were 20 mg L^−1^, solution volume was 100 mL, and pH was 5.0). **c** Comparison of the Ag^+^ maximum adsorption capacity (*q*_m_) and selectivity coefficient (*k*) of amorphous MoO_x_ with other Ag^+^-adsorbents. **d** Removal efficiency of amorphous MoO_x_ for Ag^+^ at different pH. **e** Removal efficiency of Ag^+^ in a binary mixed solution of Ag^+^ and Cu^2+^ (the Cu^2+^/Ag^+^ mass ratio is 1:1, 10:1, and 100:1). **f** Removal efficiency of amorphous MoO_x_ for Ag^+^ in the presence of different organic acids (initial Ag^+^ concentration was 20 mg L^−1^, solution volume was 100 mL, and initial organic acid concentration was 200 mg L^−^^1^). All the error bars in this figure represent the standard deviation of the data after two measurements. Source data are provided as a Source Data file.

The adsorption isotherm (Fig. 1a) of the amorphous MoO_x_ demonstrates that the Ag^+^ uptake capacity is increased promptly with increasing equilibrium concentrations (initial concentrations ranging from 10 to 250 mg L^−1^). For a comparison, bare FTO achieves scarcely any Ag^+^ removal within 120 min (Supplementary Fig. 7). Two typical isothermal adsorption models (Langmuir and Freundlich models) were used to fit the above data (Supplementary Table 2), and the adsorption process fits better with the Langmuir model (*R*^2^ = 0.948). Then, the Langmuir model was employed to predict the theoretical capacity of MoO_x_ to remove Ag^+^, and the model has been widely applied to the isotherm data associated with the reduction/oxidation removal/recovery of As^3+^, Cr^3+^, Au^3+^, Hg^2+^, and Ag^+^^35–39^. Interestingly, the maximum adsorption capacity (*q*_m_) was calculated to be as high as 2605.91 mg g^−1^, implying that amorphous MoO_x_ possesses an excellent application potential in the field of Ag^+^ recovery.

High adsorbent selectivity is essential for recovering heavy metal ions from actual wastewater^40^. Binary and multi-metal ions mixed solutions were used to evaluate the selectivity of amorphous MoO_x_. In the binary mixed solution, the Ag^+^ uptake efficiency is 49.66–473.33 times that of other metal ions at equal initial concentrations (inset of Fig. 1b). In the multi-metal ions mixed solution, as expected, 98.98% of Ag^+^ is removed; nevertheless, the removal efficiencies of other metal ions are all below 0.3% (Fig. 1b). Besides, the distribution coefficients (*k*_d_) of the amorphous MoO_x_ were also calculated (Supplementary Table 3), and the *k*_d_ value for Ag^+^ is 6437.40 mL g^−1^, which is 4 × 10^4^ to 2 × 10^5^ times greater than that of other metal ions (only 0.03–0.15 mL g^−1^). Then, the selectivity coefficients (*k*) of Ag^+^ relative to the other coexisting ions were also obtained, and the values are all higher than 4.2 × 10^4^, suggesting that the amorphous MoO_x_ possesses an excellent selectivity towards Ag^+^ adsorption.

The *q*_m_ and *k* of the amorphous MoO_x_ for Ag^+^ uptake were compared with those of previously reported Ag^+^-adsorbents (including polymers, ion-imprinted adsorbents, resins, and organic adsorbents, Supplementary Table 4). Figure 1c shows the relationship between *q*_m_ and *k*. The *q*_m_ value of the amorphous MoO_x_ is 2.99–73.45 times higher than that of previously reported adsorbents (35.48–872.63 mg g^−1^). In addition to the ultrahigh adsorption capacity, impressively, the amorphous MoO_x_ has the largest *k* value (mean value 119866.1), which is approximately 131.82 times that of other adsorbents (only 909.30 of average value). It is well known that ion-imprinted materials possess excellent selectivity; however, the *k* value of amorphous MoO_x_ is 1113.58 times that of the ion-imprinted adsorbent (only 107.64). The ultrahigh adsorption capacity and remarkable selectivity endow the amorphous MoO_x_ with a strong ability to recover Ag from actual Ag-polluted wastewater.

### Anti-interference performance

For amorphous MoO_x_, the uptake efficiencies of Ag^+^ are all over 99% under weakly acidic and neutral conditions (Fig. 1d). In addition, the pH value decreases after Ag^+^ adsorption (e.g., from 6.0 to 3.8), suggesting a proton release process^41^. Notably, even under strongly acidic conditions (Fig. 1d), amorphous MoO_x_ still maintains an exceptional Ag^+^ removal efficiency (94.6%), demonstrating superior acid-resistance performance. Furthermore, the selectivity for Ag^+^ was also investigated under high concentrations of other coexisting metal ions. Cu^2+^ was selected for comparison due to the concomitance feature with Ag^+^. High concentrations of Cu^2+^ (the Cu^2+^/Ag^+^ mass ratio up to 100:1) show almost no influence on Ag^+^ adsorption (Fig. 1e), which offers a great possibility of extracting Ag from Cu-containing industrial wastewater. In addition, the effect of salinity was tested using different concentrations of NaNO_3_ (from 0 to 1.0 M). More than 97.2% of Ag^+^ is removed under each concentration of NaNO_3_, even up to 1.0 M (Supplementary Fig. 8), showing that salt ions in water exhibit little influence on Ag^+^ recovery. Finally, to investigate the interference of dissolved organic matter in wastewater, different small-molecule organic acids with concentrations up to 200 mg L^−1^ were also studied. Surprisingly, there was no significant change in the removal efficiency of Ag^+^, which remained over 97.5% (Fig. 1f). Amorphous MoO_x_ with a nonporous structure and low specific surface area (17.58 m^2^ g^−1^) prevents the adsorption of small organic impurities and thus provides a strong resistance to organic pollutant interference^42^. Therefore, amorphous MoO_x_ possesses strong adaptability performance, providing feasibility for the selective recovery of Ag^+^ from complex wastewater.

### Self-enhancing recovery mechanism

**Fig. 2:**
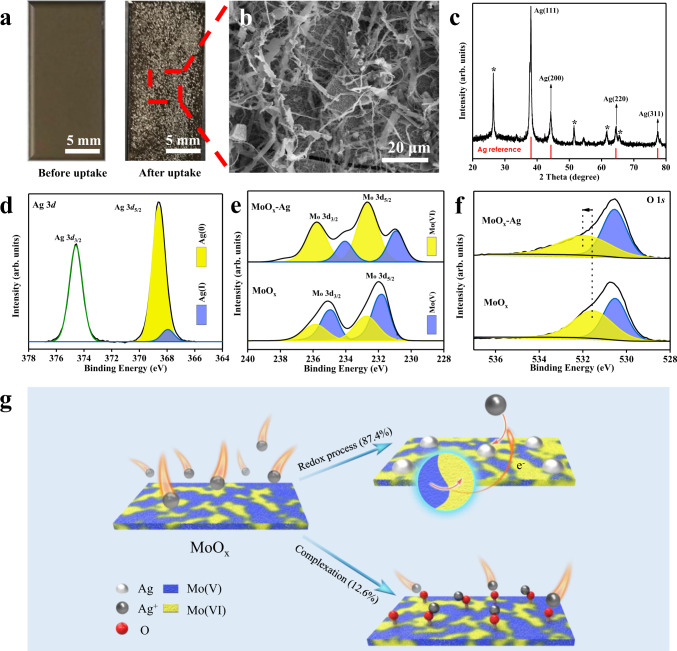
Macroscopic mechanism of Ag^+^ reduction on MoO_x_. **a** Optical photographs of FTO loaded with amorphous MoO_x_ before and after Ag^+^ adsorption. **b** SEM image revealing strip structure Ag particles. **c** XRD pattern showing the characteristic peaks of metallic Ag (the diffraction peaks marked with “*” originate from the FTO substrate). **d** High-resolution XPS spectra of the Ag 3*d* orbital. High-resolution XPS spectra of **e** Mo 3*d* and **f** O 1*s* orbitals for MoO_x_ before and after Ag^+^ uptake. **g** Macroscopic schematics of Ag^+^ capture. Mo(V) on MoO_x_ acts as an electron donor to reduce most of Ag^+^, and a small amount of Ag^+^ is trapped by complexation with MoO_x_. Source data are provided as a Source Data file.

After the Ag^+^ adsorption process, plenty of tiny silver-white particles accumulate on the surface of the amorphous MoO_x_ (Fig. 2a). It is suspected that Ag^+^ is reduced and deposited on MoO_x_. As expected, four typical peaks at 2*θ* = 38.2°, 44.5°, 64.5°, and 77.5°, which are consistent with the crystal phase of metallic Ag appear in the XRD pattern (Fig. 2c)^43^. Wide scan XPS spectra (Supplementary Fig. 9) also suggest the presence of metallic Ag. Furthermore, the high-resolution XPS spectra of Ag 3*d* were analyzed in detail. Figure 2d shows that the deconvoluted peaks centered at 374.59 eV and 368.50 eV are assigned to Ag 3*d*_5/2_ and Ag 3*d*_3/2_, respectively^44^. The Ag 3*d*_5/2_ peak was further deconvolved into doublets, which are assigned to Ag(0) and Ag(I)^45,46^. Among the total silver captured by MoO_x_, the proportion of Ag(0) is as high as 87.4%, suggesting that reductive deposition is the dominant way for MoO_x_ to capture Ag^+^. Interestingly, the reduced Ag particles form a strip structure and are clustered together (Fig. 2b). This occurs because incipient Ag nanoparticles possess better conductivity (Nyquist plots shown in Supplementary Fig. 10), which could act as an “e-bridge” for transferring electrons from MoO_x_ to reduce more outer Ag^+^, and thus, the strip structure was formed^47^. As shown in the EDS mapping images (Supplementary Fig. 11), Ag and Mo are uniformly distributed, suggesting that the reduction deposition of Ag is highly related to Mo on MoO_x_. Moreover, no significant difference was observed for Ag^+^ under light and dark conditions (Supplementary Fig. 12), demonstrating that the reduction of Ag^+^ is not caused by photo-excited electrons of MoO_x_^48^.

To further illuminate the redox process between MoO_x_ and Ag^+^, the high-resolution Mo 3*d* XPS spectra were also analyzed after Ag^+^ adsorption (Fig. 2e). The deconvoluted peaks centered at 230.90 eV and 234.08 eV correspond to Mo(V) 3*d*_5/2_ and 3*d*_3/2_, respectively (the other two peaks at 232.70 and 235.88 eV are assigned to Mo(VI))^49^. Interestingly, the relative content of Mo(V) decreased from 71.9 to 28.4% during Ag^+^ deposition; simultaneously, the Mo(VI) content increased from 28.1 to 66.7%. The changes in Mo(V) and Mo(VI) content can be attributed to the oxidation conversion from Mo(V) to Mo(VI), in which the Mo(V) species transfer electrons to realize the reduction of Ag^+^. Notably, after Ag^+^ uptake, the peak position of Mo(V) shifted to lower binding energies by almost 0.9 eV (i.e., from 234.96 to 234.08 eV for Mo(V) 3*d*_3/2_). This is owing to the interfacial electron transfer between Ag^+^ and Mo(V) species, which could induce the partial structural evolution of external Mo-O^50,51^, resulting in a larger binding energy shift. Additionally, high-resolution O 1*s* XPS spectra before and after Ag^+^ adsorption were also analyzed (Fig. 2f). During Ag^+^ adsorption, the peak position of Mo-O shifted from 531.53 to 531.90 eV, which could result from the complexation interaction between Ag^+^ and O^52^. Based on the above analysis, Ag^+^ uptake by amorphous MoO_x_ can be mainly attributed to the redox process in which most Ag^+^ (87.4%) was reduced to metallic Ag by the Mo(V) species, at the same time, Mo(V) was converted to Mo(VI) on MoO_x_. The other part of Ag^+^ (12.6%) was captured by MoO_x_ through complexation to form Mo-O-Ag and ion exchange reaction with H^+^^53^.

**Fig. 3:**
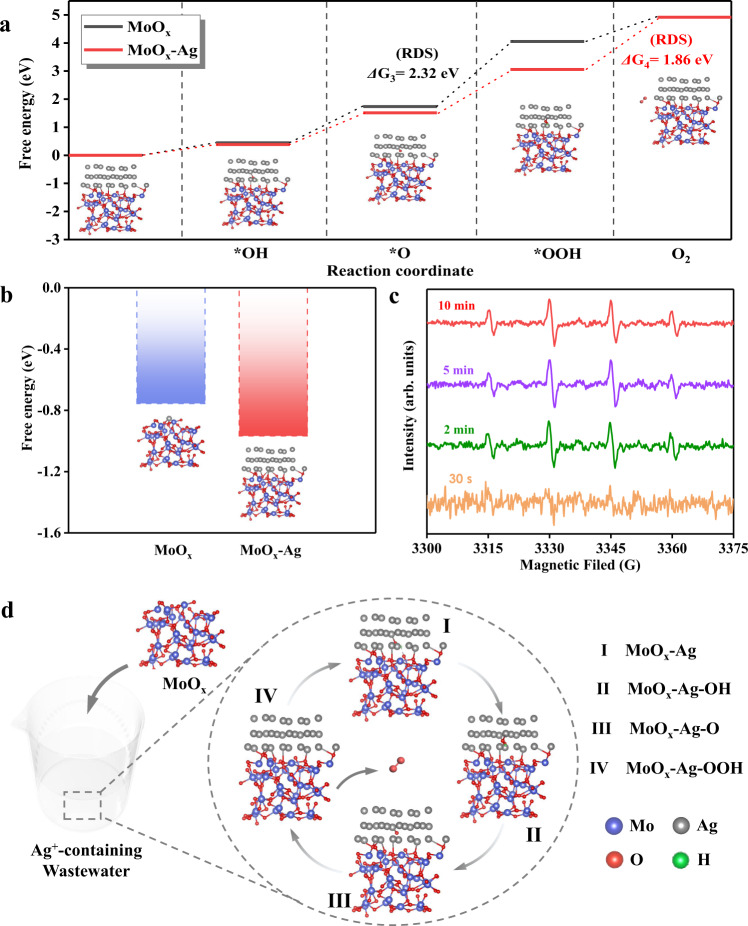
Understanding the self-enhancing reductive deposition mechanism for Ag^+^. Free energy of **a** the intermediary process of the reduction deposition for Ag^+^ on MoO_x_ and MoO_x_-Ag (rate-determining step, RDS), and **b** Ag^+^ reduction deposition on MoO_x_ and MoO_x_-Ag. The insets show the optimized MoO_x_ and intermediates on MoO_x_-Ag. The data were obtained with DFT calculations. **c** EPR spectra of Ag^+^-capture system after different capture times. **d** Schematics of the self-enhancing reduction mechanism of Ag^+^ on MoO_x_. The reductive deposition of Ag^+^ on MoO_x_ is accompanied by a series of intermediate processes (I–IV). All material structure drawings were created by VESTA^57^. Source data are provided as a Source Data file.

Although the reductive deposition of Ag^+^ by MoO_x_ has been proven to be the main way for Ag^+^ capture on a macroscopic scale, the amount of Mo(V) is lower than that deposited metallic Ag on MoO_x_. Considering the inherent excellent electron transport and catalytic ability of silver, it was suspected that the metallic Ag deposited on MoO_x_ may lower the energy barrier for the reduction deposition of Ag^+^ and strengthen the subsequent reduction process. In this respect, we performed density functional theory (DFT) calculations, simulating the process of Ag^+^ capture by MoO_x_ and MoO_x_-Ag (MoO_x_ deposited metallic Ag). The DFT calculations show that multiple intermediate processes may occur during the reductive deposition of Ag^+^ on MoO_x_ (Fig. 3a), which is consistent with previous reports^54^. The free energies of *OH, *O, and *OOH (* indicates the active sites on MoO_x_ and MoO_x_-Ag) on MoO_x_-Ag are greater than those on MoO_x_, which suggests that the deposited Ag improves the surface activity of MoO_x_. Moreover, the rate-determining step on MoO_x_ is the transformation of *O → *OOH with a free energy of 2.317 eV, but the rate-determining step on MoO_x_-Ag changed from *O → *OOH to *OOH → O_2_. This is attributed to the stability of *OOH enhanced by the deposition of metallic Ag, which in turn improved the reactivity of the entire intermediate process^55^. Importantly, MoO_x_-Ag exhibits a lower free energy of Ag^+^→*Ag than MoO_x_ (−0.756 eV and −0.967 eV, respectively), indicating that Ag^+^ was more easily reduced and deposited on MoO_x_-Ag than MoO_x_ (Fig. 3b). The above results explain why the amount of deposited Ag is higher than the amount of Mo(V) oxidized on MoO_x_. During the capture of Ag^+^, the intermediate reactions on the surface of MoO_x_ can provide additional electrons for subsequent Ag^+^ reduction, and the deposited Ag on MoO_x_ lowers the energy barrier for further reductive deposition of Ag^+^, resulting in more Ag^+^ being reduced. In-situ EPR was employed to discern the hydroxyl radicals in the Ag^+^ capture system. Figure 3c shows that hydroxyl radicals produced obvious signals when the adsorption time was 2 min, 5 min, and 10 min. This observation proves that the reductive deposition of Ag^+^ on MoO_x_ is accompanied by the above-mentioned intermediate processes and verifies the reliability of the DFT calculation results. Besides, DFT calculation results show that the intermediate process on the MoO_x_ surface generates protons. This is consistent with the pH change observed in the solution after the adsorption experiment, which also indirectly certifies the accuracy of the DFT calculations.

Integrated with the above experimental and theoretical evidence, it can be concluded that the reduction deposition of Ag^+^ on MoO_x_ is a self-enhancing process. Figure 3d shows the self-reinforcing reduction mechanism. In Ag-containing wastewater, Mo(V) on MoO_x_ acts as an electron donor to donate electrons to reduce Ag^+^. After metallic Ag is deposited on MoO_x_, the activity of MoO_x_ is enhanced, the whole reduction system is more stable, and the energy barrier of subsequent silver ion deposition is lowered, which results in more silver ions being captured. Furthermore, this unique mechanism can well explain the excellent Ag^+^ capture performance of MoO_x_. The redox potential of Mo(VI)/Mo(V) is approximately 0.53 V vs. SHE^56^ (measured value 0.37 V, Supplementary Fig. 13), much lower than the standard electrode potential of Ag(I)/Ag(0) (about 0.80 V), which enables the redox process spontaneously occur. Nevertheless, competing ions (i.e., Cu^2+^, Co^2+^, Ni^2+^, Cd^2+^, and Cr^3+^) possess lower redox potentials (Supplementary Fig. 1); hence, amorphous MoO_x_ showed excellent selectivity towards Ag^+^ uptake. Notably, according to the Nernst equation, Cu^2+^ ions can be removed only if the Cu^2+^ concentration is about 1.13 × 10^8^ times that of Ag^+^ (Supplementary Method 4). Consequently, this self-enhancing reduction mechanism could endow amorphous MoO_x_ with excellent selectivity and strong anti-interference ability for Ag^+^ recovery from real samples of complex actual wastewater.

### Closed-loop recovery of metallic Ag

**Fig. 4:**
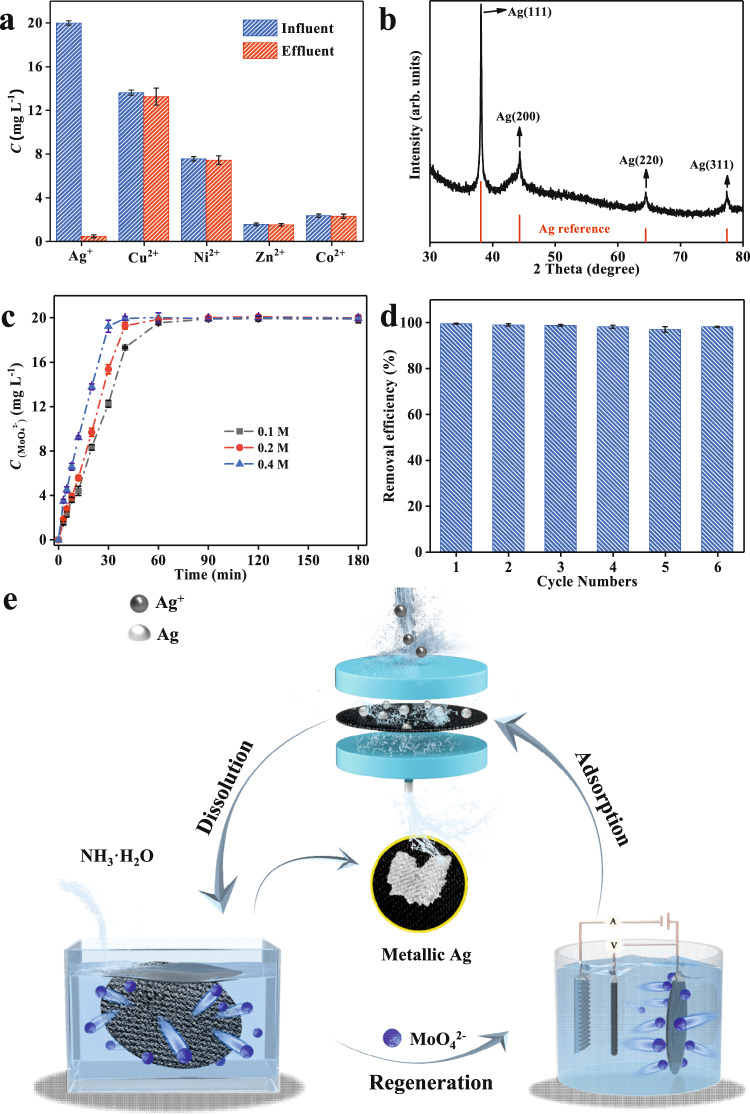
Closed-loop recovery for Ag. **a** Concentration changes of different metal ions in actual wastewater samples before and after the flow-through device (solution volume was 100 mL and pH was 5.56). **b** XRD pattern of carbon cloth-loaded amorphous MoO_x_ after Ag^+^ uptake. **c** Recovery of MoO_4_^2^^−^ using different concentrations of NH_3_·H_2_O (0.1–0.4 M). **d** Removal efficiency of the regenerated amorphous MoO_x_ for Ag^+^ (initial Ag^+^ concentration was 20 mg L^−1^, solution volume was 100 mL, and pH was 5). **e** Schematics of closed-loop recovery of metallic Ag. MoO_x_ undergoes a cycle of adsorption, dissolution, and regeneration to achieve a closed-loop recovery of Ag. The devices represent the flow-through device (top), the dissolution cell (left), and the electrochemical reactor (right), respectively. All the error bars in this figure represent the standard deviation of the data after two measurements. Source data are provided as a Source Data file.

Flow-through recovery tests were conducted to investigate the performance of Ag^+^ capture from real Ag^+^-containing wastewater samples via the amorphous MoO_x_. The one-step CV electrodeposition method enables amorphous MoO_x_ to be loaded on various substrates with different sizes (Supplementary Fig. 14). Low-cost carbon cloth with multiple channels was used for supporting the amorphous MoO_x_ to act as a membrane implanted in the designed flow-through device (Supplementary Fig. 15). The actual Ag^+^-containing electroplating wastewater (including COD, Cu^2+^, Ni^2+^, Zn^2+^, Co^2+^, NO_3_^−^, and so on) was pumped through the flow-through device, and the concentrations of effluent were measured. As shown in Fig. 4a, the concentration of Ag^+^ after filtration was 0.42 mg L^−1^, which can reach the national integrated wastewater discharge standard (GB8978-1996), and the recovery rate reached 0.35 mg L^−1^ min^−1^. Other competing metal ions show no obvious concentration change, which is consistent with the above batch test results. The XRD pattern further verifies the formation of metallic Ag (Fig. 4b), and the purity of Ag recovered from the complex wastewater was as high as 99.79% (details in Supplementary Method 5).

A dilute NH_3_·H_2_O solution was employed to recover metallic Ag and realize the regeneration of amorphous MoO_x_. After dissolution, the metallic Ag particles remained and were collected at the bottom, enabling Ag recovery. The UV-vis spectra of the regeneration solution are identical to those of the original (NH_4_)_2_Mo_2_O_7_ (Supplementary Fig. 16), which indicates that the regeneration solution can be used to synthesize amorphous MoO_x_. The concentrations of recovered MoO_4_^2^^−^ all reach 20 mg L^−1^ in dilute NH_3_·H_2_O solution (0.1–0.4 M), and the recovery rate increased with NH_3_·H_2_O concentration (Fig. 4c). As expected, amorphous MoO_x_ was prepared through the above CV electrodeposition technique from the obtained MoO_4_^2^^−^ solution. The regenerated adsorbent can still maintain over 97.1% Ag^+^ uptake performance even after the fifth cycle (Fig. 4d). Figure 4e shows the schematic of the above closed-loop process of metallic Ag recovery and adsorbent regeneration. Furthermore, an economic analysis of Ag^+^ recovery was also conducted, and repairing 1 t of this actual Ag^+^-containing electroplating wastewater resulted in a profit of $552.98 (Supplementary Fig. 17). All these results demonstrate that the amorphous MoO_x_ can not only recover metallic Ag from complex Ag^+^-containing wastewater with superior selectivity and ultrahigh anti-interference ability but also realize the sustainable circulation of adsorbents.

## Discussion

In summary, we proposed a strategy for the closed-loop recovery of Ag^+^ based on amorphous mixed-valence MoO_x_. The primary mechanism for Ag^+^ capture on MoO_x_ was demonstrated to be a self-reinforcing reductive deposition. Mo(V) on MoO_x_ acts as an electron donor to trigger the reduction of Ag^+^ and the intermediate processes of oxygen precipitation, which can provide additional electrons for the reduction of Ag^+^. After the deposition of metallic Ag, the energy barrier for Ag^+^ reduction is lowered, causing more Ag^+^ to be reduced on MoO_x_. Owing to this distinctive mechanism, MoO_x_ exhibits an ultrahigh capture capacity (2605.91 mg g^−1^), which is among the highest values reported to date, as well as excellent selectivity for Ag^+^. The recovery of metallic Ag from actual Ag-containing electroplating wastewater was achieved with a purity of up to 99.79%, showing the excellent practical application potential of MoO_x_. Interestingly, the used amorphous MoO_x_ can be dissolved in the ammonia solution, and the generated MoO_4_^2^^−^ can be recycled as the raw material for the re-synthesis of MoO_x_. Compared to conventional solvent desorption, the closed-loop regeneration strategy of MoO_x_ is waste-free and the capture performance for Ag^+^ exhibits almost no loss. The Ag^+^ closed-loop recovery method proposed in this work is potentially meaningful for recycling adsorbents and developing sustainable adsorption technology.

## Methods

### Reagents and materials

Ammonium molybdate tetrahydrate ((NH_4_)_6_Mo_7_O_24_·4H_2_O, ≥ 99%) was purchased from J&K Scientific Ltd. (Shanghai, China). Silver nitrate (AgNO_3_, ≥99%) was obtained from Aladdin Co., Ltd. (Shanghai, China). Other reagents and materials are described in Supplementary Method 1.

### Preparation of amorphous MoO_x_

Amorphous MoO_x_ was synthesized by one-step electrodeposition using the cyclic voltammetry (CV) method. First, 100 mL freshly mixed solution containing 2 mM (NH_4_)_6_Mo_7_O_24_·4H_2_O (0.2472 g, 0.2 mmol) and 0.5 M Na_2_SO_4_ (7.1 g, 0.05 mol) was prepared to act as the precursor. Then, the CV electrodeposition was carried out under magnetic stirring on a CHI 760E electrochemical workstation (CH Instruments, Shanghai) with a three-electrode system (Supplementary Fig. 2a): A conductive glass FTO as the working electrode, a Pt net as the counter electrode, and a saturated Ag/AgCl electrode as the reference electrode. The potential range was performed between –1.29 and –0.09 V at a scan rate of 50 mV s^−1^. The film was visible after five scans, and after 25 scans, the heights of the two redox peaks approached saturation (Supplementary Fig. 2b). The characterization methods of amorphous MoO_x_ are presented in Supplementary Method 2.

### Selective adsorption

The adsorption isotherm for Ag^+^ was investigated with increasing concentrations in batch experiments (details in Supplementary Method 3). For selective adsorption, the coexisting metal ions (i.e., Ni^2+^, Cu^2+^, Cr^3+^, Cd^2+^, and Co^2+^ ions) of the Ag-polluted wastewater were used. A binary solution (composed of Ag^+^ and the other coexisting ion) and a multi-metal solution consisting of the above six metal ions were used to evaluate the selectivity, respectively. The concentration of each metal ion in the binary and multi-metal solution was 20 mg L^−1^. A piece of amorphous MoO_x_ (10 mg) was soaked into 100 mL of the above two mixed solutions for 10 h under evenly stirred conditions at room temperature (25 ± 2 °C). Then, the solution was filtered through a syringe filter, and the filtrates were analyzed by an atomic absorption spectrometer (AAS, ContrAA 700, Analytik Jena, Germany) to determine the concentrations. Finally, the distribution coefficient (*k*_d_, mL g^−1^) values were calculated using the following Eq. (1):

1 $${k}_{{{{{{\rm{d}}}}}}}=\frac{\left({C}_{0}-{C}_{{{{{{\rm{e}}}}}}}\right)\,V}{{mC}_{{{{{{\rm{e}}}}}}}}$$

where *C*_0_ (mg L^−^^1^) and *C*_e_ (mg L^−1^) are initial and final concentrations, respectively; *V* (mL) and *m* (g) is solution volume and adsorbent mass, respectively.

The adsorption selectivity for Ag^+^ in the presence of other metal ions can be expressed by a selectivity coefficient (*k*) as following Eq. (2):

2 $$k=\frac{{k}_{{{{{{{\rm{d}}}}}}}_{1}}}{{k}_{{{{{{{\rm{d}}}}}}}_{2}}}$$

where *k*_d1_ and *k*_d2_ are the distribution coefficient (mL g^−1^) of Ag^+^ and another competing metal ion, respectively.

### Anti-interference tests

The effects of different environmental factors (i.e., pH, salinity, and organic pollutants) on the adsorption performance of the amorphous MoO_x_ were studied. First, 20 mg L^−1^ AgNO_3_ aqueous solution was prepared, and HNO_3_ or NaOH solution was used to adjust the solution pH to the range of 1.0–6.0 (higher pH value would lead to Ag^+^ precipitation). Amorphous MoO_x_ was soaked into the above solution for 10 h, and the filtered solution after adsorption was analyzed by AAS. Using the same procedure, salt tolerance of Ag^+^ adsorption was tested with the addition of NaNO_3_ solution at different concentrations of 0.001, 0.01, 0.1, and 1.0 M. Moreover, different small-molecule organic acids (succinic acid, citric acid, tartaric acid, malic acid, and oxalic acid) were also tested, and the concentration of the organic acids was 200 mg L^−1^.

### Theoretical calculations

All the calculations were performed within the framework of the density functional theory (DFT) as implemented in the Vienna Ab initio Software Package (VASP 5.4.4) code within the Perdew–Burke–Ernzerhof (PBE) generalized gradient approximation and the projected augmented wave (PAW) method. The cutoff energy for the plane-wave basis set was set to 450 eV. The Brillouin zone of the surface unit cell was sampled by Monkhorst–Pack (MP) grids, with a k-point mesh density of 2π × 0.04 Å^−1^ for structures optimizations. The convergence criterion for the electronic self-consistent iteration and force was set to 10^−5^ eV and 0.01 eV/Å, respectively. The PBE+U approach was applied to calculations of the electronic structure of MoO_x_ and MoO_x_-Ag in this work which can partly reduce the underestimation of the electronic band gap and the excessive tendency to delocalize the electron density. In this work, we set the Hubbard parameter to U − J = 4 eV for Mo. A vacuum layer of 15 Å was introduced to avoid interactions between periodic images.

### Flow-through recovery tests

A flow-through device was designed to realize the Ag^+^ recovery from actual Ag^+^-containing electroplating wastewater (Supplementary Fig. 3). The device possesses a chamber with an inner diameter of 3.0 cm and a depth of 2.0 cm. The amorphous MoO_x_ was loaded onto the surface of carbon cloth (HCP331N, 0.25 mm in thickness, 3.0 cm in radius) through the above CV method using the three-electrode system. Three pieces of amorphous MoO_x_ modified carbon cloths were stacked in the device chamber and served as a functional filter to trap Ag^+^. The actual Ag^+^-containing wastewater was obtained from Nanchang Electroplating Industrial Zone (located at N28°37'84.12”, E116°24'08.45”), and the wastewater characteristics are shown in Supplementary Table 1.

### Closed-loop recovery of metallic Ag

The amorphous MoO_x_ after Ag^+^ adsorption was dissolved using different concentrations of NH_3_·H_2_O solution (0.1–0.4 M). The concentration of MoO_4_^2^^−^ was measured using a Thermo Scientific ICAP-Q inductively coupled plasma mass spectrometer (ICP-MS, Waltham, MA) and an ultraviolet-visible spectrophotometer (U-3900H, Hitachi, Japan). For the recyclability test, Ag-deposited amorphous MoO_x_ was immersed in 0.2 M NH_3_·H_2_O solution for 2 h. Then, metallic Ag was filtered and recovered, and the MoO_4_^2^^−^ in the filtrate was collected and used as raw materials for the electrochemical synthesis of amorphous MoO_x_. The regenerated adsorbents were reused in the next cycle of the above flow-through recovery system.

## Supplementary information

Supplementary Information

Peer Review File

## Data Availability

The data that supports the findings of this study are available in the article and Supplementary information file and available from the authors upon request.  Source data are provided with this paper.

## Source data

Source Data
