# Supplementary material for: Mixed-valence molybdenum oxide as a recyclable sorbent for silver removal and recovery from wastewater
Source: Nat Commun. 2023 Mar 13;14:1365. doi: 10.1038/s41467-023-37143-2 (PMC10011435; doi:10.1038/s41467-023-37143-2)
Supplement: Supplementary file 1 — Supplementary Information [file 41467_2023_37143_MOESM1_ESM.pdf]

Supplementary Information

Mixed-valence molybdenum oxide as a recyclable sorbent for silver removal and recovery from wastewater

Penghui Shao[1], Ziwen Chang[1], Min Li[2,*], Xiang Lu[1], Wenli Jiang[3], Kai Zhang[1], Xubiao Luo[1], Liming Yang[1,*]

[1] National-Local Joint Engineering Research Center of Heavy Metals Pollutants Control and Resource Utilization, Nanchang Hangkong University, Nanchang 330063, P. R. China

[2] Department of Chemical Engineering, Chongqing University of Science and Technology, Chongqing 401331, P. R. China

[3] Key Laboratory of Environmental Biotechnology, Research Center for Eco-Environmental Sciences, Chinese Academy of Sciences, Beijing 100085, P. R. China

* Corresponding author

E-mail address: yangliming0809185@126.com (L. M. Yang)
limin1406@163.com (M. Li)

Supplementary Methods 1

Reagents and Materials

Copper nitrate trihydrate ($Cu(NO_3)_2 \cdot 3H_2O$, ≥99%), Cobalt nitrate hexahydrate ($Co(NO_3)_2 \cdot 6H_2O$, ≥99%), nickel nitrate hexahydrate ($Ni(NO_3)_2 \cdot 6H_2O$, ≥98%), cadmium nitrate tetrahydrate ($Cd(NO_3)_2 \cdot 4H_2O$, ≥99%), zinc nitrate hexahydrate ($Zn(NO_3)_2$, ≥99%), chromium (III) nitrate nonahydrate and sodium sulfate anhydrous (Na_2SO_4, ≥99%) were supplied by Xilong Chemical Co., Ltd. (Guangdong, China). Succinic acid (≥99.5%), citric acid (10 wt %), DL-tartaric acid (≥99.9%), DL-malic acid (≥99) and oxalic acid (≥98) were purchased from Aladdin Co., Ltd. (Shanghai, China).

Supplementary Methods 2

Characterization Methods

The morphology of the as-prepared sample was observed by scanning electron microscopy (SEM, Shimadzu SSX-550). The N_2 sorption isotherms were measured using a Micromeritics TriStar II 3020 at 77 K. X-ray photoelectron spectroscopy (XPS) measurements were performed on an Axis Ultra (Kratos, XSAM800) XPS spectrometer with Al target (1486.6 eV) X-ray source. X-ray diffraction (XRD) spectra were obtained using a graphite-monochromated Co Kα radiation (λ = 0.179 nm) on a D8 ADVANCE system (40 kV, 40 mA, Bruker, Madison, WI). EPR spectroscopy (Burk EMXplus spectrometer, Germany) was used to study the generation of reactive free radicals.

Supplementary Methods 3

Isothermal Adsorption

The adsorption isotherm for Ag^+ was investigated with increasing concentrations (i.e., 10, 25, 50, 75, 100, 150, 200 and 250 mg L^{-1}) in batch experiments. Different concentrations of a 100 mL $AgNO_3$ aqueous solution were added into different beakers. Then, a piece of amorphous MoO_x loaded FTO was soaked in the solution and evenly stirred for 10 h at room temperature (25 ± 2 °C). The solution was filtered through a syringe filter (PES, 0.45 μm filter), and the filtrates were analyzed by an atomic absorption spectrometer (AAS, ContrAA 700, Analytik Jena, Germany) to determine residual Ag^+ concentrations. Three parallel experiments were carried out to obtain the

reported data. Finally, the adsorption capacity (q_e, mg g^{-1}) was calculated according to the following Equation (1):

$$q_e = \frac{(C_0 - C_e)\ V}{m} \quad \textbf{(Equation S1)}$$

Where q_e is adsorption capacity (mg g^{-1}) at equilibrium, C_0 and C_e is initial and equilibrium concentration (mg L^{-1}) of Ag^+, respectively. V is total volume (mL) of adsorption solution, and m is adsorbent mass (mg).

Supplementary Method 4

For half-reaction $Ag^+ + e^- = Ag$ and $Cu^{2+} + 2e^- = Cu$, the redox potentials can be calculated through the Nernst equation:

$$E = E^{\theta} + \frac{RT}{nF} ln[C_m] \quad \textbf{(Equation S2)}$$

Where E is the potential of the half-reaction (V), E^{θ} is the standard potential of the half-reaction (V), R is a chemical constant (8.314 J K^{-1} mol^{-1}), T is the temperature (K), n is the electron transfer numbers, F is the Faraday constant (96485 J mol^{-1} V^{-1}), and C_m is the concentration of Ag^+ and Cu^{2+} (mol L^{-1}).

The E^{θ} of the half-reaction ($Ag^+ + e^- = Ag$) is 0.7995 V, and the E^{θ} of the half-reaction ($Cu^{2+} + 2e^- = Cu$) is 0.337 V. When the initial Ag^+ concentration is 20 mg L^{-1} (that is 1.85×10^{-4} mol L^{-1}) and the temperature is 298 K, according to the Equation S2, the E value of $Ag^+ + e^- = Ag$ is calculated to be 0.5786 V. For the half-reaction $Cu^{2+} + 2e^- = Cu$, when the E value is 0.5786V, the calculated initial concentration of Cu^{2+} is about 2.26×10^9 mg L^{-1}, which is 1.13×10^8 times that of the initial concentration of Ag^+.

Supplementary Methods 5

The purity of the recovered metallic Ag can be calculated by the following formula:

$$p = \frac{m_1}{m} \quad \text{(Equation S3)}$$

Where the p is the purity of recovered Ag (%), m_1 is the mass of recovered Ag (mg), and m is the total mass of recovered metals (including Ag^+, Cu^{2+}, Ni^{2+}, Zn^{2+}, and Co^{2+}, mg).

The values of m_1 and m are obtained from the difference in the concentration of heavy metal ions in the wastewater and regenerative solutions, where the volume of the

wastewater and regenerative solutions is 100 mL and 50 mL, respectively. Through calculation, the values of m_1 and m are 1.955 mg and 1.959 mg, respectively. Therefore, the purity of silver recovered from wastewater is as high as 99.79%.

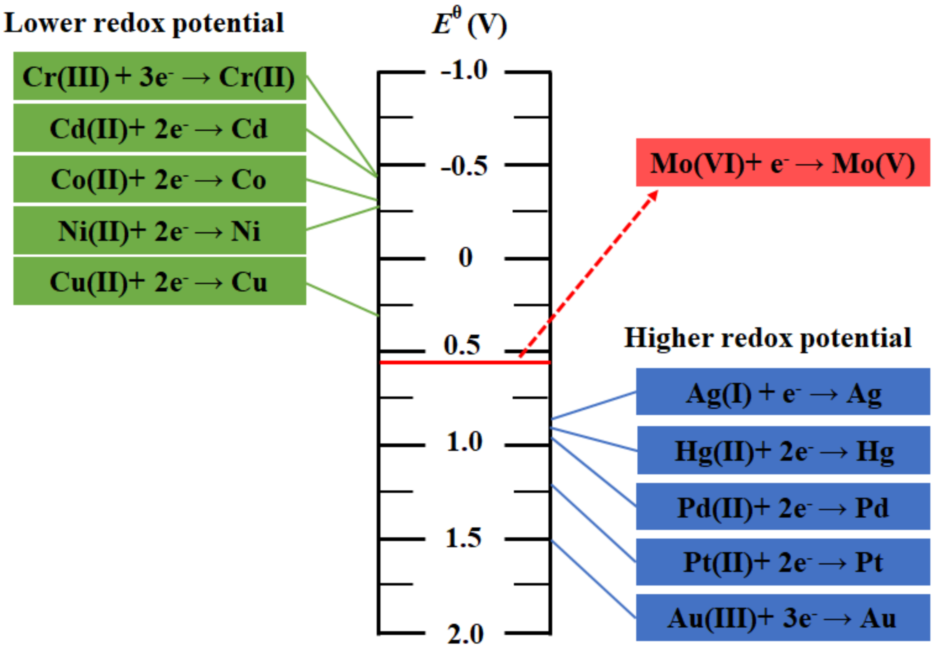

Supplementary Figure 1. Standard redox potential diagrams for different metal ions (*vs*. SHE).

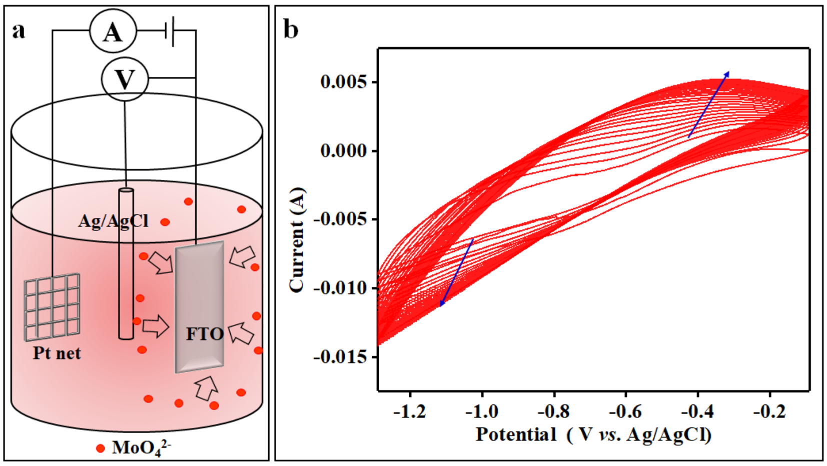

Supplementary Figure 2. Electrochemical synthesis of amorphous MoO_x. a Schematic representation for electrochemical deposition of amorphous MoO_x film on FTO. **b** Cyclic voltammogram of FTO upon the electrochemical deposition of amorphous MoO_x film.

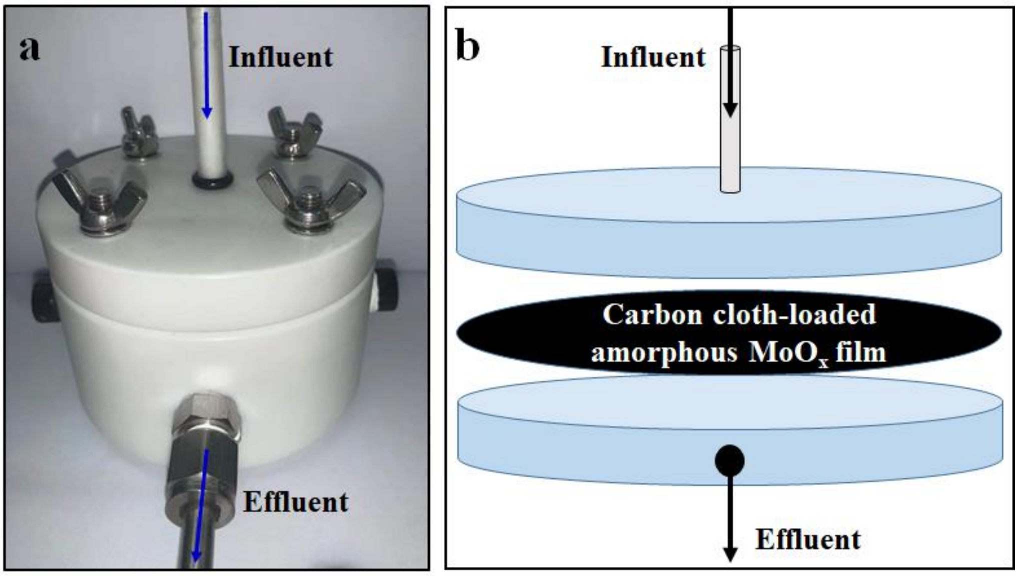

Supplementary Figure 3. Flow-through device. a Optical picture. **b** schematic of the flow-through device.

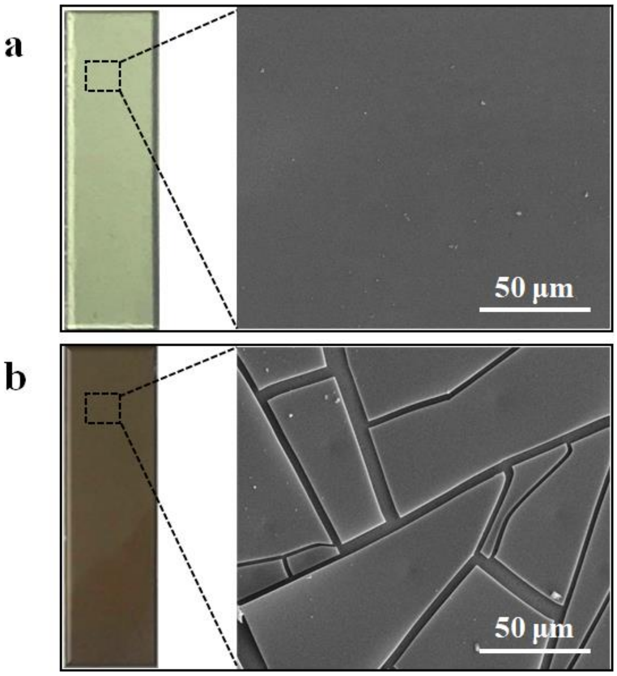

Supplementary Figure 4. Optical and SEM images of bare FTO and amorphous MoO_x film. a Bare FTO. **b** Amorphous MoO_x film.

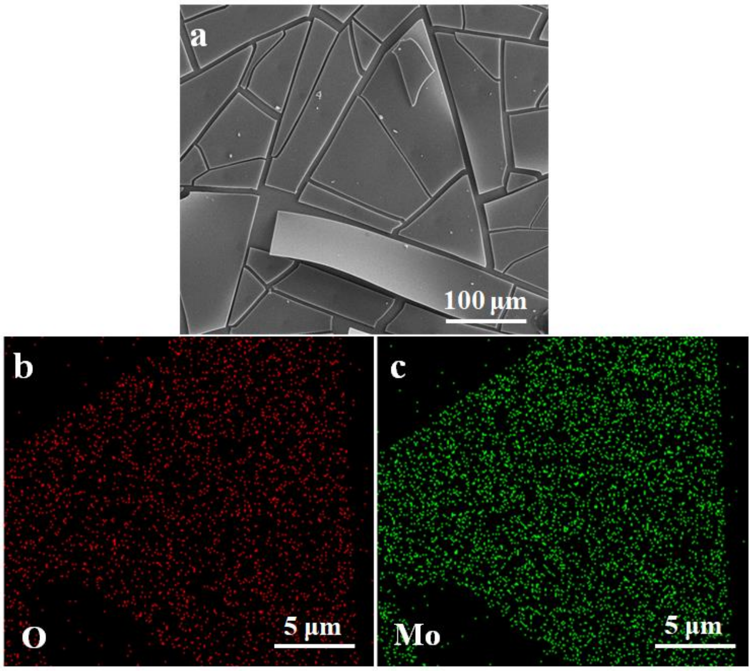

Supplementary Figure 5. SEM image and the corresponding EDS elemental mapping of MoO_x. a SEM images of the amorphous MoO_x film. Corresponding EDS elemental mapping of a selected region of amorphous MoO_x film for **b** O and **c** Mo.

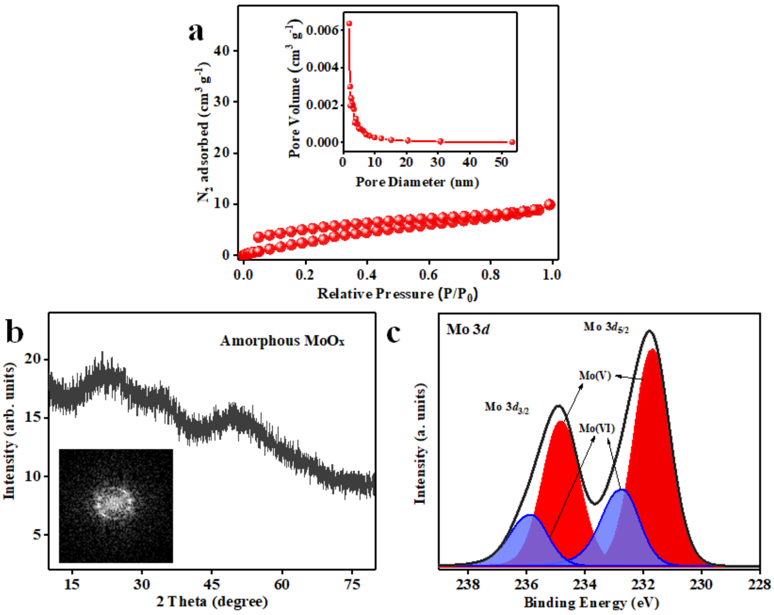

Supplementary Figure 6. The BET, XRD and XPS characterization of MoO_x. a N_2 adsorption-desorption isotherm (inset is the pore size distribution). **b** XRD pattern of the amorphous MoO_x film (inset showing the SAED pattern). **c** High-resolution Mo 3*d* XPS spectra.

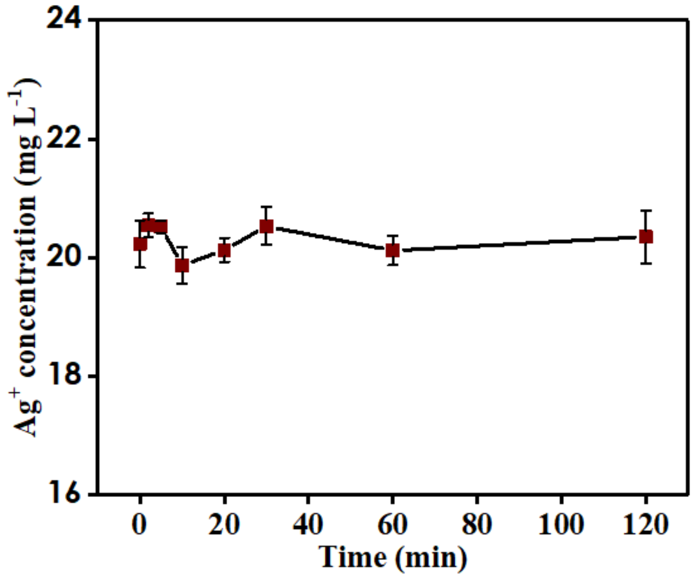

Supplementary Figure 7. The curve of Ag^+ concentration with time after immersion of FTO conductive glass. All the error bars in this figure represent the standard deviation of the data after 2 measurements.

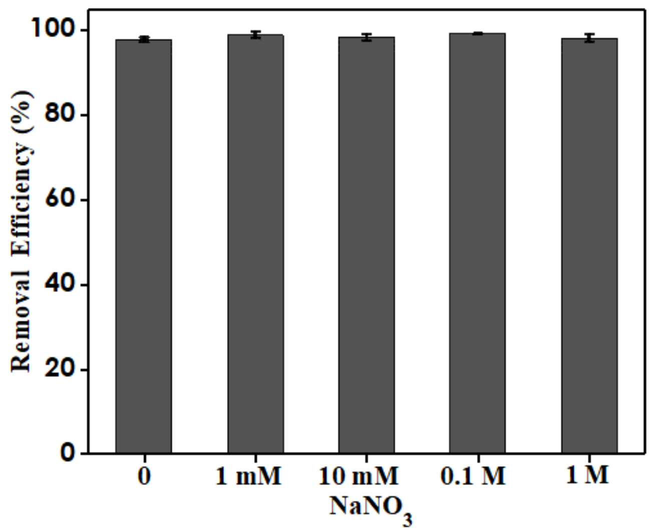

Supplementary Figure 8. Removal efficiency of amorphous MoO_x for Ag^+ in different concentrations of nitrate. All the error bars in this figure represent the standard deviation of the data after 2 measurements.

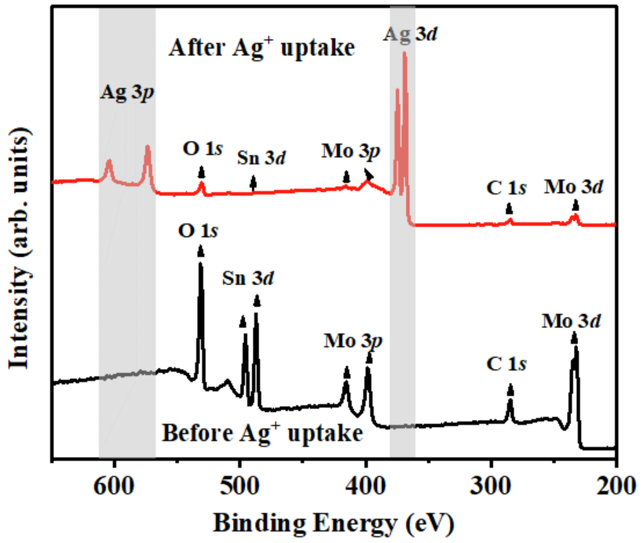

Supplementary Figure 9. Wide scan XPS spectra of the amorphous MoO_x film before and after Ag^+ uptake. Sn and C elements come from the FTO substrate.

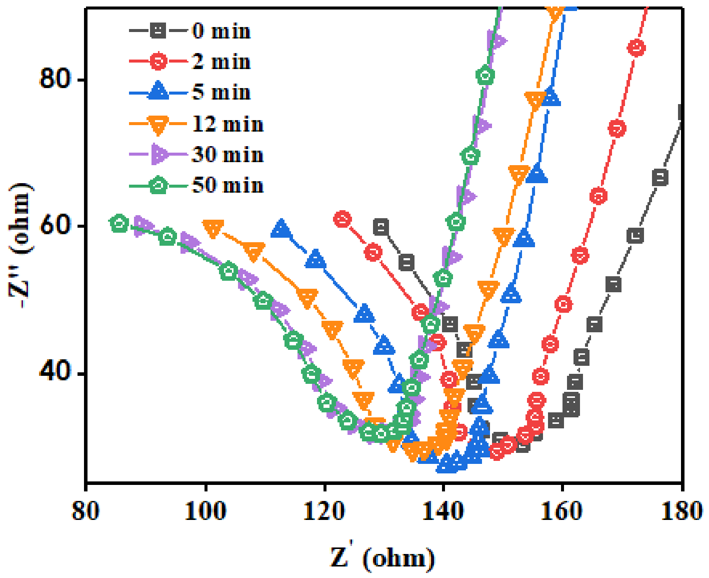

Supplementary Figure 10. Nyquist plots of MoO_x after capture Ag^+ for a different time in 0.1 mol L^{-1} $NaNO_3$ solution with an open potential.

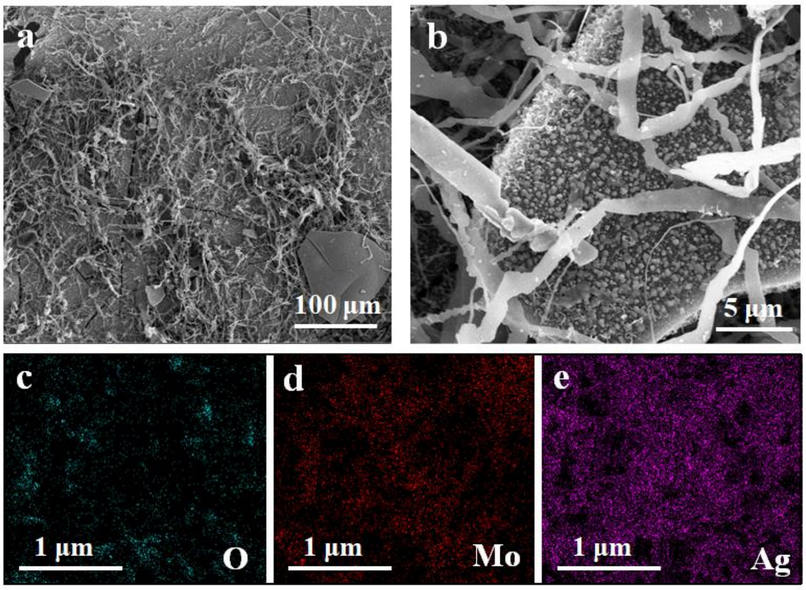

Supplementary Figure 11. SEM images and the corresponding EDS elemental mapping of MoO_x after Ag^+ uptake. a, b SEM images of Ag-containing FTO-loaded amorphous MoO_x film with different magnifications revealing strip-like Ag particles. The corresponding EDS elemental mapping of a selected region of amorphous MoO_x for **c** O, **d** Mo and **e** Ag.

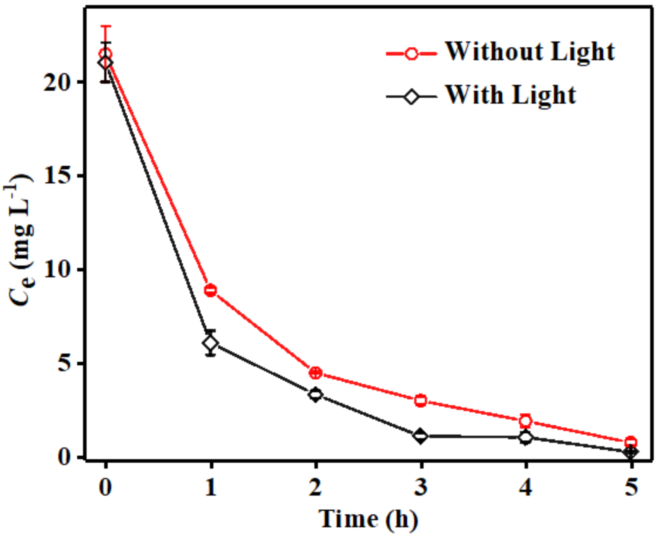

Supplementary Figure 12. Ag^+ concentration changes in the solution over time after FTO-loaded amorphous MoO_x film immersed with or without light. A xenon lamp was used as light source. All the error bars in this figure represent the standard deviation of the data after 2 measurements.

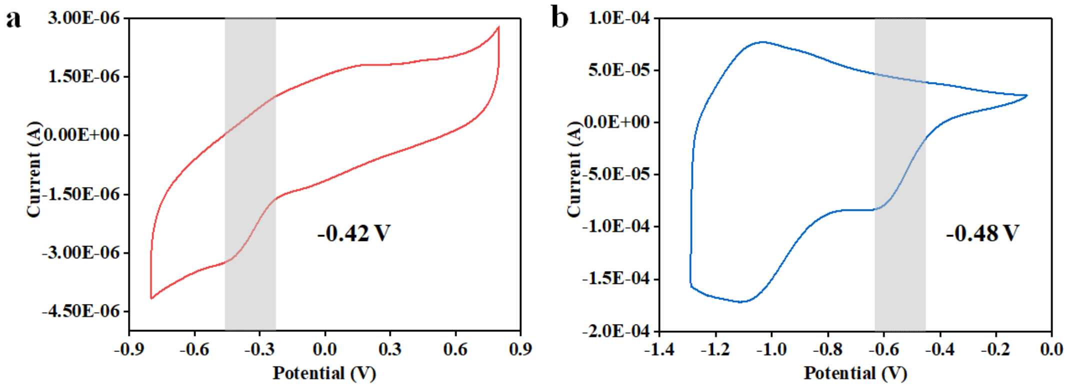

Supplementary Figure 13. Electrochemical characterizations of the redox potential of MoO_x. a Cyclic voltammograms of amorphous MoO_x film in N_2-saturated 0.1 M $NaNO_3$ solution at a scan rate of 50 mV s^{-1}. **b** Cyclic voltammograms of glassy carbon electrode (GCE) in a freshly mixed solution containing 0.2 mmol $(NH_4)_6Mo_7O_{24}$ $4H_2O$ and 0.05 mol Na_2SO_4 at a scan rate of 50 mV s^{-1}; All electrochemical characterizations were performed in a three-electrode system: the counter electrode was a Pt mesh, and the reference electrode was a Ag/AgCl electrode.

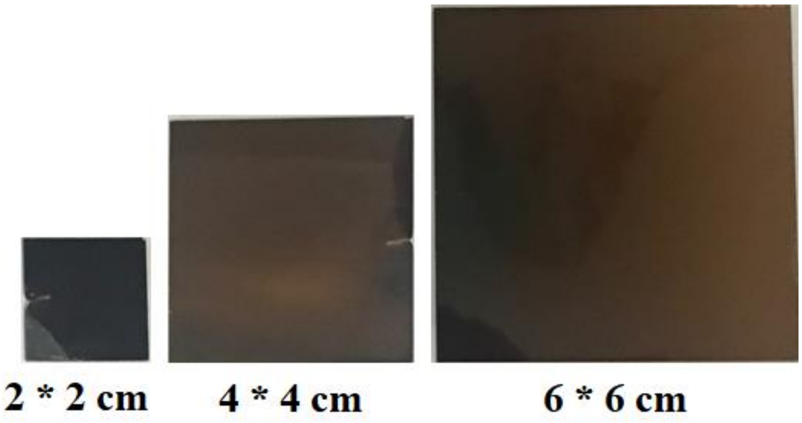

Supplementary Figure 14. Optical images of amorphous MoO_x film on different size FTO.

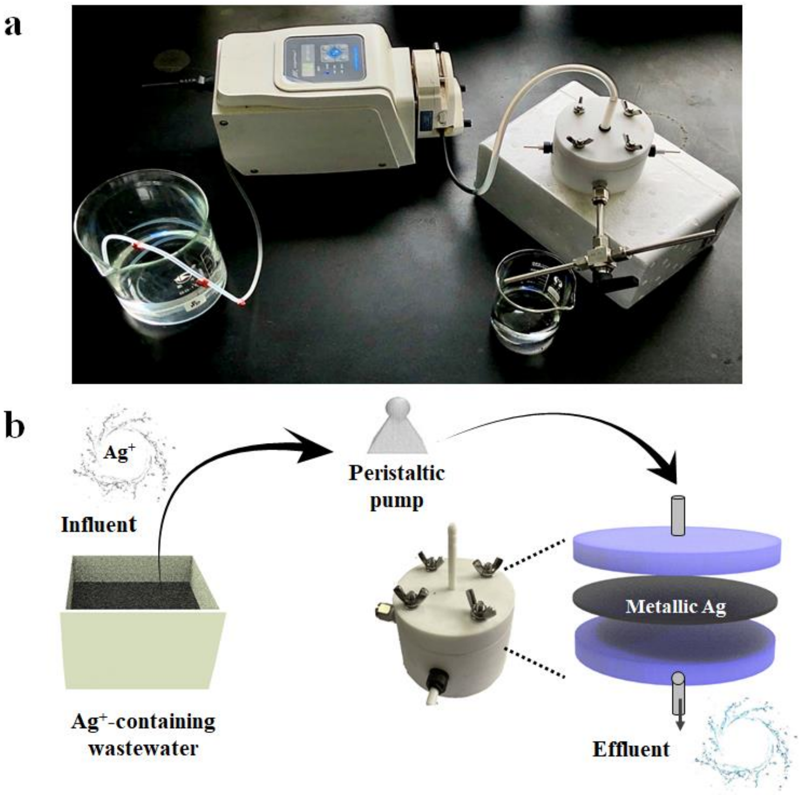

Supplementary Figure 15. Flow-through system for capturing Ag^+. a Optical picture of the flow-through system. **b** Schematics of the flow-through system.

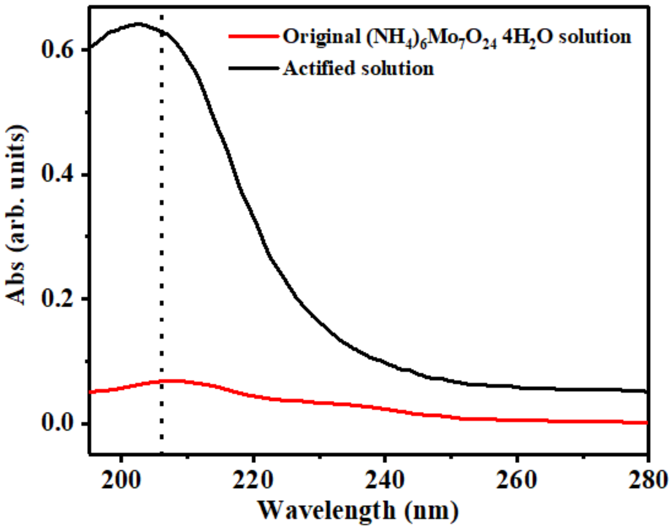

Supplementary Figure 16. UV-vis spectra of $(NH_4)_6Mo_7O_{24}\cdot 4H_2O$ solution and actified solution of amorphous MoO_x film using $NH_3\cdot H_2O$ solution.

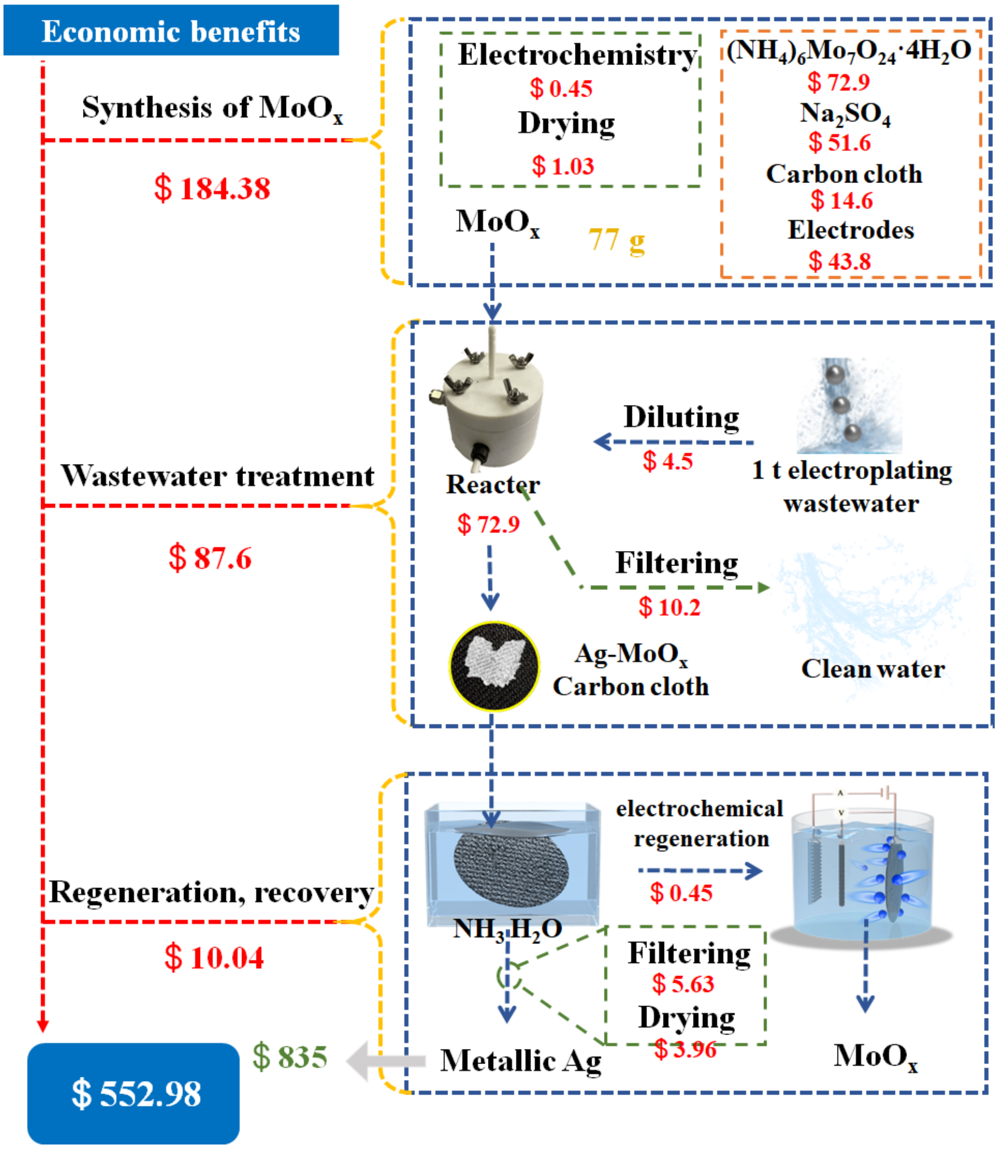

Supplementary Figure 17. Economic analysis of Ag^+ recovery from 1 t Ag- containing wastewater.

Supplementary Table 1. The main composition and content of the Ag^+-containing actual wastewater.

**pH**	**COD**	**TOC**	**Ag^+**	**Cu^{2+}**	**Ni^{2+}**	**Zn^{2+}**	**Co^{2+}**
5.56	500 mg L^{-1}	142.9 mg L^{-1}	200 mg L^{-1}	150.2 mg L^{-1}	75.3 mg L^{-1}	15.1 mg L^{-1}	23.1 mg L^{-1}

Supplementary Table 2. The parameters of the Langmuir fitting and Freundlich fitting.

Langmuir isotherm			Freundlich isotherm		
q_m(mg g^{-1})	k_l	R^2	1/n	k_f	R^2
2605.91	0.5499	0.965	0.2067	802.99	0.912

Langmuir model: $q_e = \frac{q_m k_l C_e}{1 + k_l C_e}$ **(Equation S4)**

Freundlich model: $q_e = k_f C_e^{1/n}$ **(Equation S5)**

Where C_e and q_e are the concentration (mg L^{-1}) and adsorbing capacity (mg g^{-1}) of the Ag^+ at adsorption equilibrium; k_l is the Langmuir constant; k_f is the Freundlich constant and q_m is the maximum adsorption capacity (mg g^{-1}).

Supplementary Table 3. Selective adsorption parameters of amorphous MoO_x film toward six common metal ions (initial concentration 20 mg L^{-1}).

**Mixed ions**	**M^{n+} removal (%)**	**k_d (mL g^{-1})**	***k***
Ag^{+}	98.98	6437.40	
Ni^{2+}	0.23	0.15	41886.47
Cu^{2+}	0.12	0.08	83869.5
Cr^{3+}	0.04	0.03	241305.9
Co^{2+}	0.14	0.09	71430.09
Cd^{2+}	0.06	0.04	160838.4

Supplementary Table 4. Maximum adsorption capacity (q_m) and selectivity coefficient (k) for the adsorption of Ag^+ onto various adsorbents.

Adsorbents	q_m (mg g^{-1})	k	Competing ions	Ref.
PAR	651	744	K^+, Co^{2+}, Pb^{2+}, Cu^{2+}, Ni^{2+}, Cd^{2+}	1
Fe_3O_4@SiO_2@TiO_2-IIP	35.475	29.96	Li^+, Co^{2+}, Cu^{2+}, Ni^{2+}	2
Ag-TCM	531.8	2.385	Cd^{2+}, Zn^{2+}, Pb^{2+}, Cu^{2+}	3
TCE@PP	172	99	Cu^{2+}, Zn^{2+}, Ni^{2+}, Pb^{2+}	4
TCP	560.91	60	Cu^{2+}	5
R-en	121.89	463.96	Cu^{2+}, Pb^{2+}, Cd^{2+}, Zn^{2+}, Ca^{2+}	6
CS/PVA	125	5.5	Cu^{2+}, Ni^{2+}	7
BA-PGMA	157.05	50.075	Cu^{2+}, Zn^{2+}, Ni^{2+}, Co^{2+}	8
PAM-TL	145.2	316.31	K^+, Co^{2+}, Pb^{2+}, Cu^{2+}, Ni^{2+}, Cd^{2+}	9
T-PGMA	217.17	11436.14	Co^{2+}, Ni^{2+}, Zn^{2+}	10
TSC-CC	872.63	11.41	Hg^{2+}	11
ITG-OCMC	156.32	485.41	Cu^{2+}	12
IGCC	89.2	118.89	Cu^{2+}	13
Agim75	120.4	3.75	Cu^{2+}	14
PS-TMT	187.1	556.43	Ni^{2+}, Zn^{2+}, Pb^{2+}, Cu^{2+}, Cr^{3+}	15
TU-PVA	66.93	993.87	Ni^{2+}, Zn^{2+}, Pb^{2+}, Cu^{2+}	16
ATT-SNPs	124.52	80.96	Ni^{2+}, Zn^{2+}, Co^{2+}, Ge^{4+}	17
MoO_x	2605.91	1.2×10^5	Ni^{2+}, Cu^{2+}, Cr^{3+}, Co^{2+}, Cd^{2+}	This work

Abbreviations: PAR, poly-allylrhodanine; Fe_3O_4@SiO_2@TiO_2-IIP, Ag(I)-imprinted thiol-functionalized polymer; Ag-TCM, magnetic thiourea-chitosan; TCE@PP, thia-crown ethers polypropylene membrane; TCP, thioether/carboxyl-functionalized polymer; R-en, chitosan/amine; CS/PVA, chitosan/poly(vinyl alcohol); BA-PGMA, poly(glycidyl methacrylate with carboxymethyl groups; PAM-TL, polymerization of thiolactone functionalized acrylamide; T-PGMA, poly(glycidyl methacrylate with trithiocyanuric acid; TSC-CC, cyanuric-thiosemicarbazid chelating resin; ITG-OCMC, Ag^+-imprinted thiourea/glutaraldehyde grafted O-carboxymethyl chitosan; IGCC, Ag^+-imprinted chitosan gel beads; Agim75, Ag^+-imprinted chitosan hydrogels; PS-TMT, trimercaptotriazine-functionalized polystyrene chelating resin; TU-PVA, poly(vinyl alcohol) modified with thiourea adsorbent; ATT-SNPs, modifying silica nanoparticles with 3-amino-5-mercapto-1,2,4-triazole

Supplementary References

1. Yin, X., Shao, P., Ding, L., Xi, Y., Zhang, Kai., Yang, L., Shi, H., Luo, X. Protonation of rhodanine polymers for enhancing the capture and recovery of Ag^+ from highly acidic wastewater. *Environ. Sci.-Nano*. **6**, 3307-3315 (2019).
2. Yin, X., Long, J., Xi, Y., Luo, X. Recovery of silver from wastewater using a new magnetic photocatalytic ion-imprinted polymer. *ACS Sustain. Chem. Eng.* **5**, 2090-2097 (2017).
3. Fan, L., Luo, C., Lv, Z., Lu, F., Qiu, H. Removal of Ag^+ from water environment using a novel magnetic thiourea-chitosan imprinted Ag^+. *J. Hazard. Mater*. **194**, 193-201 (2011).
4. Fissaha, H. T., Nisola, G. M., Burnea, F. K., Lee, J. Y., Koo, S., Lee, S. P., Hern, K., Chung, W. J. Synthesis and application of novel hydroxylated thia-crown ethers as composite ionophores for selective recovery of Ag^+ from aqueous sources. *J. Ind. Eng. Chem.* **81**, 415-426 (2020).
5. Huang, Y., Zhao, W., Zhang, X., Peng, H., Gong, Y. Thiol-ene synthesis of thioether/carboxyl-functionalized polymers for selective adsorption of silver(I) ions. *Chem. Eng. J.* **375**, 121935 (2019).
6. Elwakeel, K. Z., El-Sayed, G. O., Darweesh, R. S. Fast and selective removal of silver(I) from aqueous media by modified chitosan resins. *Int. J. Miner. Process*. **120**, 26-34 (2013).
7. Shawky, H. A. Synthesis of ion imprinting chitosan/PVA crosslinked membrane for selective removal of Ag(I). *J. Appl. Polym. Sci.* **114**, 2608-2615 (2009).
8. Zhao, J., Wang, S., Zhang, L., Wang, C., Zhang, B. Kinetic, isotherm, and thermodynamic studies for Ag(I) adsorption using carboxymethyl functionalized poly(glycidyl methacrylate). *Polymers*. **10**, 1090 (2018).
9. Liu, X., Yang, L., Luo, X., Pei, J., Xi, Y., Liu, C., Liu, L. A novel non-imprinted adsorbent with superior selectivity towards high-performance capture of Ag(I). *Chem. Eng. J.* **348**, 224-231 (2018).
10. Xiong, C., Wang, S., Zhang, L., Li, Y., Zhou, Y., Peng, J. Selective recovery of

silver from aqueous solutions by poly(glycidyl methacrylate) microsphere modified with trithiocyanuric acid. *J. Mol. Liq*. **254**, 340-348 (2018).

11. Lin, G., Wang, S., Zhang, L., Hu, T., Peng, J., Cheng, S., Fu, L. Selective adsorption of Ag^{+} on a new cyanuric-thiosemicarbazide chelating resin with high capacity from acid solutions. *Polymers*. **9**, 568 (2017).
12. Zhang, M., Zhang, Y., Helleur, R. Selective adsorption of Ag^{+} by ion-imprinted O-carboxymethyl chitosan beads grafted with thiourea–glutaraldehyde. *Chem. Eng. J.* **264**, 56-65 (2015).
13. Zhang, M., Helleur, R., Zhang, Y. Ion-imprinted chitosan gel beads for selective adsorption of Ag^{+} from aqueous solutions. *Carbohyd. Polym*. **130**, 206-212 (2015).
14. Song, X., Li, C., Xu, R., Wang, K. Molecular-ion-imprinted chitosan hydrogels for the selective adsorption of silver (I) in aqueous solution. *Ind. Eng. Chem. Res*. **51**, 11261-11265 (2012).
15. Wang, S., Li, H., Chen, X., Yang, M., Qi, Y. Selective adsorption of silver ions from aqueous solution using polystyrene-supported trimercaptotriazine resin. *J. Environ. Sci*. **24**, 2166-2172 (2012).
16. Yang, T., Zhang, L., Zhong, L., Han, X., Dong, S., Li, Y. Selective adsorption of Ag(I) ions with poly(vinyl alcohol) modified with thiourea (TU–PVA). *Hydrometallurgy*. **175**, 179-186 (2018).
17. Fu, L., Zhang, L., Wang, S., Peng, J., Zhang, G. Selective adsorption of Ag^{+} by silica nanoparticles modified with 3-amino-5-mercapto-1, 2, 4-triazole from aqueous solutions. *J. Mol. Liq*. **241**, 292-300 (2017).
